# Extending medium-range predictability of extreme hydrological events in Europe

**DOI:** 10.1038/ncomms6382

**Published:** 2014-11-11

**Authors:** David A. Lavers, Florian Pappenberger, Ervin Zsoter

**Affiliations:** 1 European Centre for Medium-Range Weather Forecasts, Shinfield Park, Reading RG2 9AX, UK; 2 School of Geographical Sciences, University of Bristol, Bristol, BS8 1SS, UK

## Abstract

Widespread flooding occurred across northwest Europe during the winter of 2013/14, resulting in large socioeconomic damages. In the historical record, extreme hydrological events have been connected with intense water vapour transport. Here we show that water vapour transport has higher medium-range predictability compared with precipitation in the winter 2013/14 forecasts from the European Centre for Medium-Range Weather Forecasts. Applying the concept of potential predictability, the transport is found to extend the forecast horizon by 3 days in some European regions. Our results suggest that the breakdown in precipitation predictability is due to uncertainty in the horizontal mass convergence location, an essential mechanism for precipitation generation. Furthermore, the predictability increases with larger spatial averages. Given the strong association between precipitation and water vapour transport, especially for extreme events, we conclude that the higher transport predictability could be used as a model diagnostic to increase preparedness for extreme hydrological events.

## Abstract (web summary)

Extreme winter flooding has caused devastation across Europe in recent years, highlighting the need for improved forecasts. Here, the authors show that the use of water vapour transport, rather than precipitation in weather prediction models, can extend the forecast horizon by as much as 3 days.

## Graphical abstract

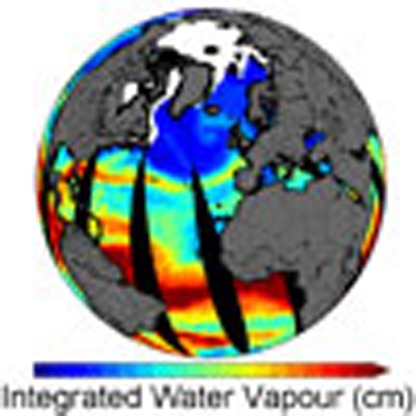

Flooding across Europe is a recurring natural hazard causing substantial socioeconomic losses^1^,^2^. It is expected that flood frequency and intensity will also change as the hydrological cycle intensifies in a warming atmosphere^3^,^4^,^5^. Previous research has shown that extreme winter European precipitation and floods are connected to water vapour transport within extratropical cyclones^6^,^7^,^8^. The water vapour transport predominantly occurs in the atmospheric river (AR) region of the warm conveyor belt of the cyclone, which is typically on the order of 400–500 km wide and 2,000 km in length^9^,^10^. Owing to the location of ARs within extratropical cyclones, the water vapour transport within them is considered *a priori* to be connected to large- (or synoptic-) scale atmospheric variability. As the synoptic-scale circulation is more predictable than the sub-synoptic variability^11^, which is generally associated with precipitation, and with the strong relationship between water vapour transport and precipitation^6^,^7^,^8^,^12^, an important question to pose is how skilful are forecasts of these variables?

Medium-range weather forecasts, often defined as having a 3–14 day lead time, can provide early warning and increased preparedness for extreme events, which in turn has societal benefits^13^. Because of the inherent errors in the initial state estimate of the atmosphere and in numerical weather prediction models, multiple realizations of the future atmosphere, or ensemble forecasting, has been developed as a way to take these into account allowing for the probability distribution of future atmospheric states to be estimated^14^. Forecasts of variables such as surface precipitation, wind or temperature can either be used directly to warn of upcoming severe events, or they can drive other models to issue alerts for hydrological extremes (for example, floods and droughts^15^), malaria occurrence, or wild forest fires^16^. For example, the European Flood Awareness System (www.efas.eu) uses medium-range ensemble forecasts to issue flood alerts across Europe^17^,^18^,^19^. The advanced warnings produced are then disseminated to the relevant hydrometeorological agencies so that preparation can be made for such events.

The aim of this study is to test the hypothesis that the vertically integrated horizontal water vapour transport (hereafter, integrated vapour transport (IVT)) is more predictable than precipitation because of the more predictable synoptic-scale processes that lie behind the IVT. Using medium-range forecasts from the European Centre for Medium-Range Weather Forecasts (ECMWF) ensemble prediction system for the winter season 2013/14 because of the widespread extreme flooding that occurred in the British Isles, we employ the potential predictability as a diagnostics tool to evaluate the forecast skill of IVT and precipitation at a range of lead times and spatial scales. The potential predictability approach assumes that the ‘forecast’ model and ‘observed’ climate systems are the same^20^,^21^, and yields an estimate of the predictability without the need for observations. This is a desirable approach, because it is free of the spatiotemporal inhomogeneities of the observational network. Our analysis shows that there is higher medium-range predictability of IVT compared with precipitation in the ECMWF numerical weather prediction model. In some European regions, including parts of England, Germany and Spain, the IVT is found to extend the forecast horizon by 3 days. Results suggest that the earlier breakdown in precipitation predictability is primarily due to uncertainty in the location of the horizontal mass convergence, a key mechanism that produces precipitation. The predictability for precipitation and IVT is also shown to increase with larger spatial averages. As precipitation is strongly connected with IVT, especially for extreme events, the higher transport predictability could be used to increase preparedness for extreme hydrological events.

## Results

### Assessment of potential predictability across Europe

**Figure 1:**
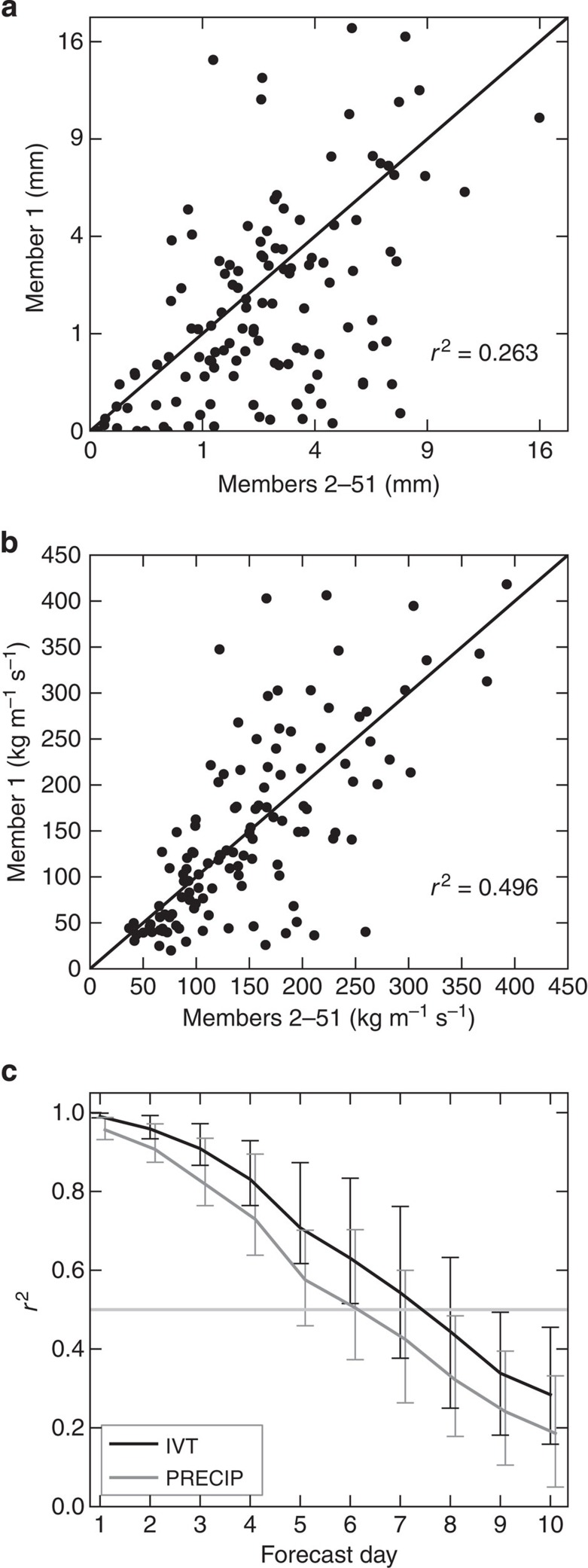
An example of the potential predictability methodology. An example of the potential predictability methodology over a 1° × 1° region in southern Britain (location given in Fig. 2a,d) on forecast day 7 for (**a**) precipitation in a square root-transformed space and (**b**) water vapour transport (IVT), when Member 1 is the observation and the average of Members 2–51 is the forecast. The 121 points are the days of the 2013/14 winter season and the black diagonal lines are the 1:1 line. (**c**) The change in potential predictability of precipitation and IVT with forecast day for southern Britain; the error bars correspond to the minimum and maximum *r*^2^-values on each forecast day. The horizontal grey line represents the benchmark skill used (*r*^2^=0.5).

**Figure 2:**
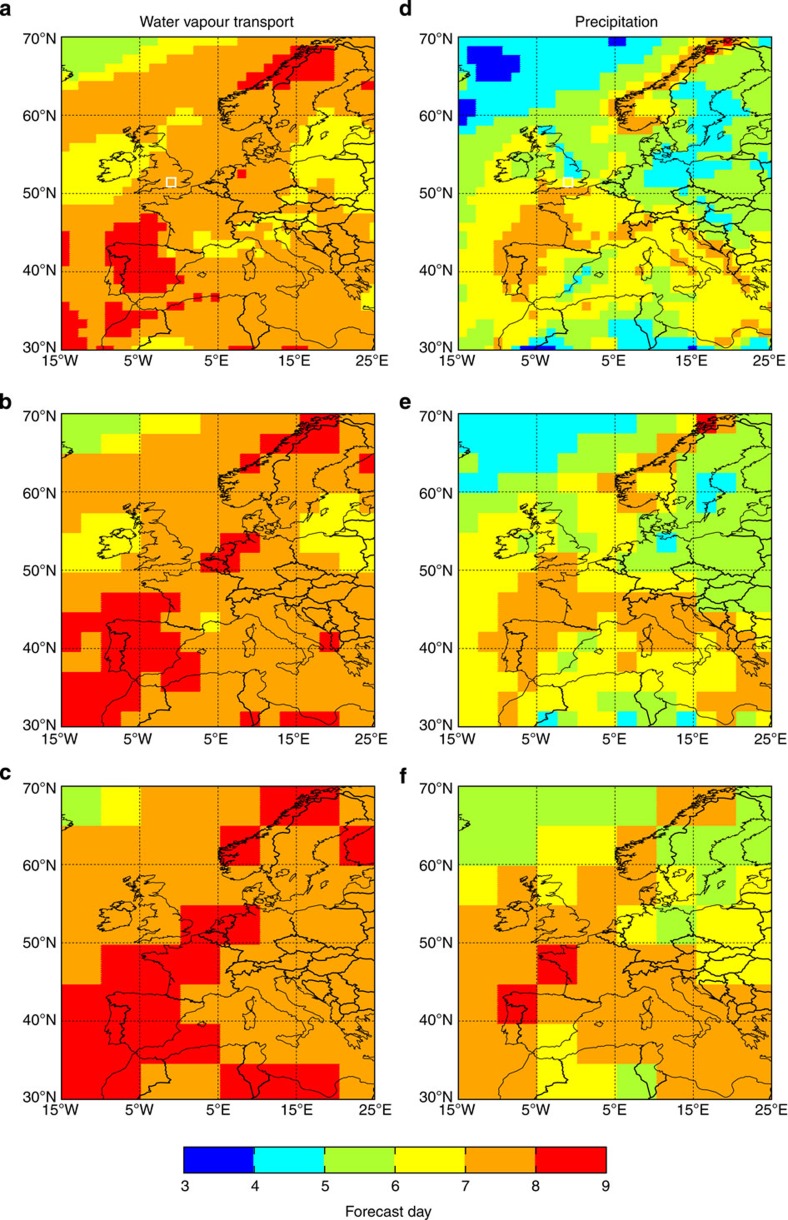
Last forecast day when the potential predictability exceeds the benchmark skill. Maps showing the last forecast day when the potential predictability exceeds an *r*^2^-value of 0.5 for (**a**–**c**) water vapour transport and (**d**–**f**) precipitation. (**a**,**d**) Spatial average of ~1° × 1° (16 model grid points); (**b**,**e**) average of 2.5° × 2.5° (81 model grid points); (**c**,**f**) average of 5° × 5° (324 model grid points). The region used for the calculations in Fig. 1 is shown by the white box in **a** and **d**.

The potential predictability approach is illustrated in Fig. 1 for a 1° × 1° area over southern Britain (centred on 51.43°N, 0.84°W; Fig. 2a,d show the location). Figure 1a,b show for the precipitation and IVT, respectively, the results on forecast day 7, where ensemble member 1 is considered as the observation and the average of members 2–51 is taken as the forecast (the ensemble mean is often used when assessing ensemble forecasts); this results in two time series of 121 values, which relate to the days in the winter season. The skill of this forecast is then calculated as the coefficient of determination (*r*^2^), or the square of the linear Pearson correlation coefficient *r* between the two time series, and Fig. 1 shows that for member 1 the IVT has higher predictability than precipitation. This method is repeated 51 times, with each member being considered as the observation, so as to obtain 51 *r*^2^-values; the average of these 51 values is the final estimate of the potential predictability. The change in potential predictability with forecast day for the area in southern Britain is displayed in Fig. 1c and the range of the 51 *r*^2^-values is shown by the error bars. It is evident that in the mean, the IVT always has higher predictability throughout the forecast period, with the largest skill difference occurring from days 5 to 7. Herein we use an *r*^2^-value of 0.5 as the benchmark to signify a skilful system, and according to this criterion the IVT in this region has an increased forecast horizon (over precipitation) of 1–2 days.

Figure 2 shows the last forecast day on which the potential predictability exceeds an *r*^2^ of 0.5 for IVT (panels a–c) and precipitation (panels d–f) for three spatial averages. The general pattern is that the highest predictability is present in the southern half of the domain (for example, over the Iberian Peninsula), whereas the lowest predictability is found to the northwest of the domain, which is likely to be due to the uncertainty associated with the location of the North Atlantic extratropical storm track. For IVT at a 1° × 1° resolution, broad areas of Europe have *r*^2^-values exceeding 0.5 out to forecast day 7, with, for example, some areas of the Iberian Peninsula and Scandinavia having this skill out to day 8. However, for precipitation the last forecast day on which the *r*^2^-values exceed 0.5 is generally less with large areas only exhibiting this skill up to forecast day 5. Strikingly parts of the British Isles and central Europe have an extra 3-day forecast horizon when considering IVT instead of precipitation. This increase in forecast skill could potentially be exploited for hydrological applications, and in particular for early warning and increased preparedness of extreme events. Furthermore, Fig. 2b,c, e and f highlight that for larger spatial averages the potential predictability increases (particularly for precipitation), which corroborates previous research^20^.

### Reasoning for higher water vapour transport predictability

We hypothesized that IVT would have higher predictability because of its relationship with synoptic-scale variability, but a further important point is to consider what mechanism is responsible for the precipitation predictability to break down more rapidly than the IVT. To address this question, we calculate the vertical integral (between 1,000 and 700 hPa) of the divergence of the water vapour transport, which by vector identity results in an advection term (moisture gradient transported by the wind) and a moisture weighted horizontal mass convergence term. We focus on the latter term (hereafter known as the moisture flux convergence (MFC)), as to leading order the total precipitation is related to the vertical integral of the MFC^22^. In Supplementary Fig. 1a–c, the MFC potential predictability is shown. Notably, at a 1° × 1° resolution the MFC potential skill is generally highest in similar regions to precipitation, that is where there are mountains, for example, in western Norway, Portugal, the Alps and the Balkans. This suggests that the fixed mountainous areas induce horizontal mass convergence, resulting in higher MFC potential predictability. In turn, higher precipitation skill occurs because the convergence being quasi-stationary causes the vertical uplift, condensation and precipitation to be more fixed in space. Conversely, over the North Atlantic Ocean and parts of central Europe the MFC potential predictability is low. This is due to larger uncertainty in the MFC location, because here the convergence and uplift is generally linked with sub-synoptic scale activity on weather fronts; hence, the precipitation predictability is also generally lower in these regions. This is partly borne out in the predictability of the 700 hPa relative humidity (Supplementary Fig. 1d–f). Where there is large uncertainty in the MFC there is mostly lower skill in the relative humidity, because broadly speaking high humidity occurs where there is vertical uplift and condensation. Although the generation of precipitation is a complicated process dependent on many factors, these results suggest that the breakdown in precipitation predictability comes from uncertainty in the location of the horizontal mass convergence, which typically varies at the sub-synoptic or mesoscale. Conversely, because of the link between IVT and the synoptic-scale extratropical cyclones, the IVT taps into the higher synoptic-scale predictability.

### ECMWF extreme forecast index for 24 December 2013

**Figure 3:**
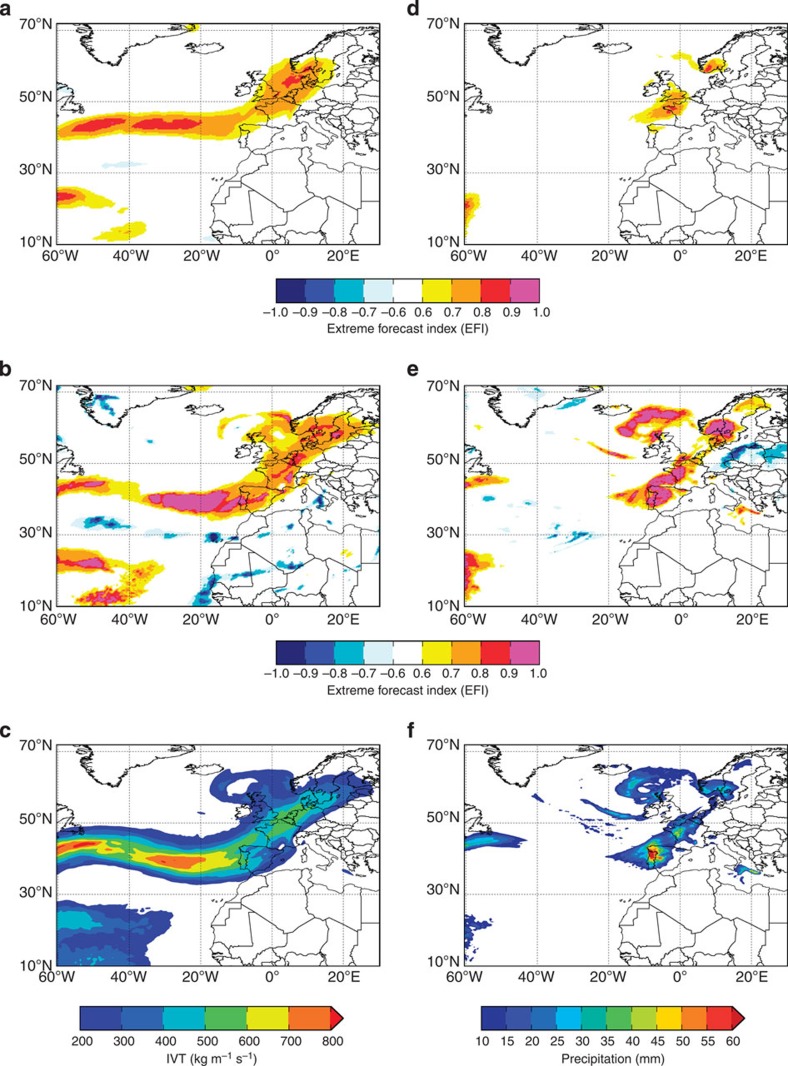
The ECMWF EFI of water vapour transport and precipitation for 24 December 2013. (**a**–**c**) Water vapour transport and (**d**–**f**) precipitation. (**a**,**d**) show the EFI on day 7 of the forecasts initialized at 00UTC 18 December 2013; (**b**,**e**) the EFI on day 1 of the forecasts initialized at 00UTC 24 December 2013 (all panels are valid for 24 December 2013). High values of EFI indicate a significant anomaly of the ensemble forecast with respect to the climatic distribution. (**c**,**f**) The water vapour transport and precipitation accumulations for the first 24 h of the ensemble control forecast from 00UTC 24 December 2013; these are used as a proxy for observations (IVT is a daily average using 00UTC and 12UTC on 24 December and 00UTC on 25 December 2013).

We investigate one possible application of the higher IVT predictability by employing the ECMWF Extreme Forecast Index (EFI)^23^,^24^ for an extreme hydrological event in Europe on 24 December 2013. (The EFI assesses how extreme the ensemble forecasts are with respect to the model climate, yielding a value between −1 and 1.) Figure 3a,d show the EFI for IVT and precipitation, respectively, on day 7 of the forecasts initialized at 00UTC 18 December 2013, while Fig. 3b,e show the EFI on day 1 of the forecasts initialized at 00UTC 24 December 2013 (all plots are valid for 24 December 2013). The intense storm and AR that affected Europe are shown in Fig. 3c,f. Although the precipitation EFI on forecast day 7 did detect an extreme event in southern Britain and western France, further east over Belgium, the Netherlands, Denmark and western Germany, no signal was found (Fig. 3d). However, by considering the IVT EFI a strong signal is shown (Fig. 3a), which maps well onto the EFI on forecast day 1 from 00UTC 24 December 2013 (Fig. 3b,e) and onto the proxy observations of IVT and precipitation (Fig. 3c,f). This increased warning corresponds to an area where higher potential predictability of about 2 days was found for IVT compared with precipitation (*cf*. Fig. 2a,d). Therefore, it should be possible to use the IVT to provide earlier warnings of extreme hydrological events because of the higher IVT predictability and due to the strong connection between water vapour transport and precipitation across Europe (Supplementary Fig. 2a).

## Discussion

The aim of this analysis was to test the hypothesis that the IVT is more predictable than precipitation because of its association with synoptic-scale variability. By estimating the potential predictability in the ECMWF ensemble forecasts for the winter 2013/14 season over Europe, we conclude that our results suggest this hypothesis to be correct. The water vapour transport does generally have the highest predictability, with, for example, some areas of England, Germany and Spain having an extended forecast horizon of 3 days compared with precipitation. It is also shown how the predictability increases with larger spatial averages. The analysis further suggests that the breakdown in precipitation predictability is due to uncertainty in the location of the horizontal mass convergence, an important mechanism for precipitation generation. This means that the errors in the precipitation forecasts are larger than those for IVT, which results in the signal being lost more rapidly for precipitation. As water vapour transport has a strong association with precipitation across Europe and in particular with heavy precipitation and floods^6^,^7^,^8^, it should be feasible to increase the skilful medium-range forecast horizon (over that of precipitation) of extreme hydrological events.

We evaluated a potential application of the enhanced medium-range predictability by applying the ECMWF EFI on the IVT and precipitation forecasts for a heavy precipitation and flood event on 24 December 2013. The results showed that the use of the IVT EFI increased warning of the upcoming extreme hydrological event in Belgium, the Netherlands, Denmark and western Germany. These regions had higher IVT potential predictability of about 2 days compared with precipitation. A detailed study would need to be undertaken to evaluate the false alarm rate of such a warning system for different applications, but we can conclude that the higher water vapour transport predictability could be used as a model diagnostic for hydrometeorological applications to increase warning and thus preparedness for extreme winter hydrological events in Europe.

## Methods

### ECMWF ensemble prediction system forecasts

The control and 50 perturbed ensemble members (out to forecast day 10) from the ECMWF forecast model were retrieved from the ECMWF Meteorological Archival and Retrieval System (MARS) for the 00UTC initialization from 1 December 2013 to 31 March 2014 (121 days). Daily total surface precipitation accumulated at 00UTC was retrieved, and the relative humidity at 700 hPa and the specific humidity *q* and the zonal and meridional (*u* and *v*) winds at 300, 400, 500, 700, 850, 925 and 1,000 hPa were retrieved at 00UTC and 12UTC during each 10 day forecast. All fields were retrieved on a reduced Gaussian grid at their native resolution of T639 (N320) and then converted onto a regular Gaussian grid (corresponding to about 0.28° × 0.28°). The daily-averaged (using 00UTC, 12UTC, and 00UTC of the next day) vertically integrated horizontal zonal and meridional water vapour transports (using layer-averaged *q*, *u* and *v* from 1,000 to 300 hPa) were then calculated in an Eulerian framework^9^ and combined into the total water vapour transport (IVT).

The vertical integral between 1,000 and 700 hPa (as the lower troposphere is the main region of moisture convergence) of the divergence of the water vapour transport was also considered, which by vector identity results in an advection term (moisture gradient transported by the wind) and a moisture weighted horizontal mass convergence term. We focus on the latter term (MFC), as to leading order the total precipitation is related to the vertical integral of MFC^22^.

### Potential predictability calculation

The potential predictability^20^,^21^ is a technique that uses the spread or variance of the ensemble members as an indicator of forecast skill, with small spread among ensemble members implying that the forecast is likely to be insensitive to the initial conditions (resulting in high skill), while large spread is likely to indicate low forecast skill. Herein, the predictability is assessed across different European regions (within 30°N-70°N, 15°W-25°E), at different spatial averages (approximately a 1° × 1°, 2.5° × 2.5° and a 5° × 5° scale) and at different forecast times (1–10 days). In this approach, for a particular region the spatial average from one ensemble member is considered as the observation, while the mean of the spatial average in the other 50 members is taken as the forecast; this results in two time series of 121 values, which relate to the days in the winter season. The skill of this forecast is then calculated as the coefficient of determination (*r*^2^) or the square of the linear Pearson correlation coefficient *r* between the two time series. (Note that a square root transformation was applied to the precipitation time series so that they more closely followed a Gaussian distribution.) This procedure is repeated 51 times, each time with a different ensemble member being considered as the observation (because all members are considered as being equally likely), so that 51 *r*^2^-values are obtained. These 51 values are then averaged to estimate the potential predictability.

### Observations of water vapour transport and precipitation

The ERA-Interim reanalysis^25^ *q*, *u* and *v* between 1,000 and 300 hPa (20 levels) were also retrieved from MARS at 00, 06, 12 and 18UTC for the extended winters (December, January, February and March) of 2009/10 to 2013/14 (5 winters). All fields were retrieved on a reduced Gaussian grid at their native resolution of T255 (N128) and then converted onto a regular Gaussian grid (corresponding to about 0.7° × 0.7°). The daily averaged (using 00UTC, 06UTC, 12UTC, 18UTC and 00UTC of the next day) vertically integrated horizontal zonal and meridional water vapour transports (using layer-averaged *q*, *u* and *v* from 1,000 hPa to 300 hPa) were then calculated in a Eulerian framework^9^ and combined into the total water vapour transport (IVT); the ERA-Interim IVT is used as a proxy for observed IVT. Daily precipitation observations for stations across Europe from the World Meteorological Organisation Global Telecommunication System were also extracted from MARS for the extended winters of 2009/10 to 2013/14. A linear Pearson correlation analysis was then employed between the precipitation (a square root transformation was applied to the precipitation time series so that they more closely followed a Gaussian distribution) and daily averaged ERA-Interim IVT time series; only stations with >50% of daily availability were considered.

### ECMWF extreme forecast index

The EFI^23^,^24^ was used to assess how extreme the ensemble forecasts were with respect to the model climate. For IVT and precipitation, the EFI was calculated for day 7 of the 51 ensemble forecasts initialized on 00UTC 18 December 2013 and for day 1 of the 51 ensemble forecasts initialized on 00UTC 24 December 2013 (verification time of 24 December 2013). The model climatology (of IVT and precipitation) used in the EFI comprises the ECMWF model hindcasts from 12, 19 and 26 December in calendar years 1993–2012 (20 years). Each hindcast has 5 members resulting in a 300-member model climate. The index has a range of −1 to 1, with −1 indicating extremely low and 1 indicating extremely high values with respect to the climatic distribution for a particular forecast variable.

## Author contributions

D.A.L. and F.P. designed the experiment. D.A.L. and E.Z. retrieved the data. D.A.L. performed the analyses. All authors were involved in the interpretation of the results. D.A.L. and F.P. wrote the paper.

## Additional information

**How to cite this article:** Lavers, D. A. *et al*. Extending medium-range predictability of extreme hydrological events in Europe. *Nat. Commun.* 5:5382 doi: 10.1038/ncomms6382 (2014).

## Supplementary Material

Supplementary Figures Supplementary Figures 1-2
