# Supplementary material for: Extending medium-range predictability of extreme hydrological events in Europe
Source: Nat Commun. 2014 Nov 11;5:5382. doi: 10.1038/ncomms6382 (PMC4242464; doi:10.1038/ncomms6382)
Supplement: Supplementary Figures — 1-2 [file ncomms6382-s1.pdf]

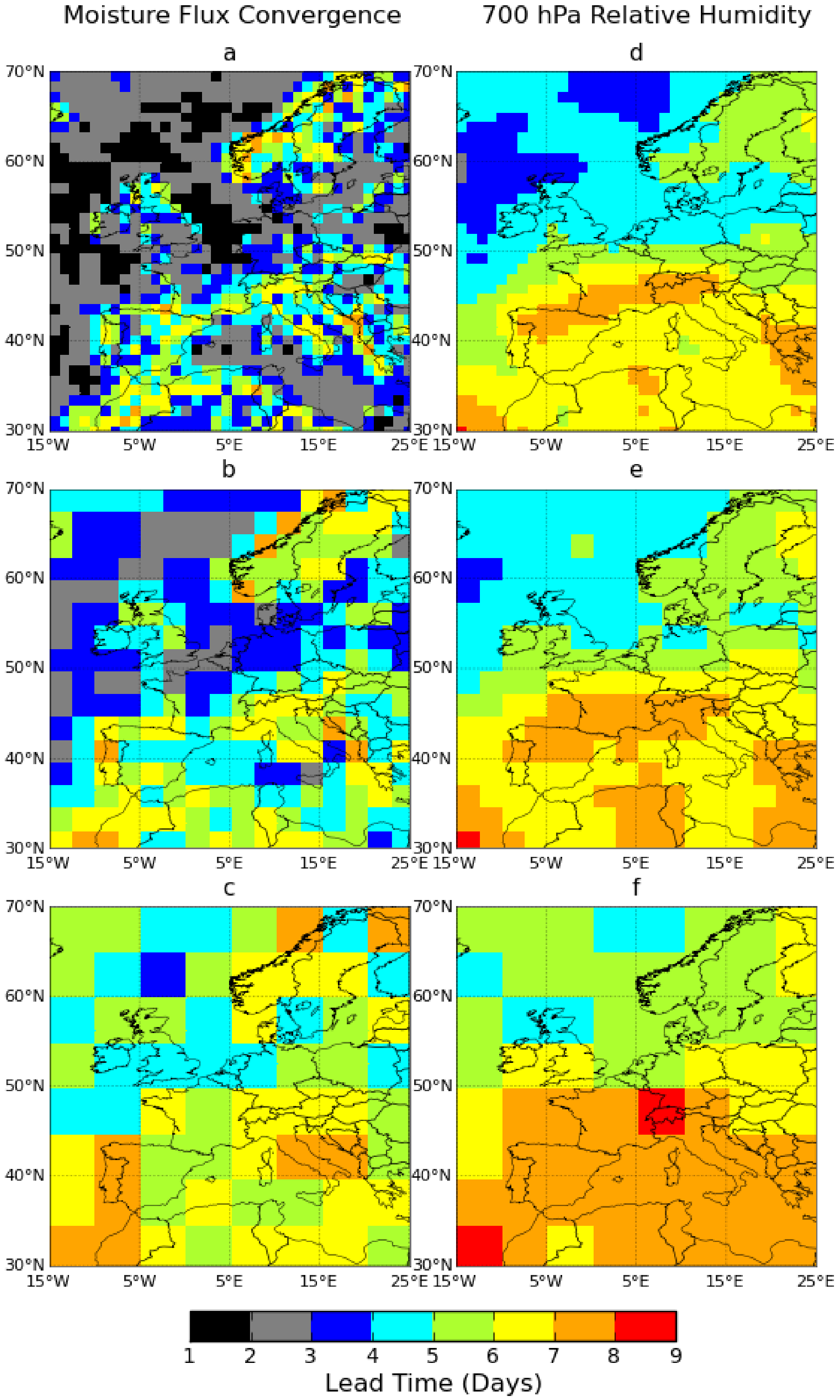
Moisture Flux Convergence
700 hPa Relative Humidity
a
b
c
d
e
f
70°N
60°N
50°N
40°N
30°N
15°W
5°W
5°E
15°E
25°E
1
2
3
4
5
6
7
8
9
Lead Time (Days)

Supplementary Figure 1: Maps showing the last forecast day when the potential predictability exceeds an r^2 value of 0.5 for (a-c) moisture flux convergence (MFC), and (d-f) 700 hPa relative humidity. Panels (a) and (d) correspond to a spatial average of approximately 1°×1° (16 model grid points), panels (b) and (e) to an average of 2.5°×2.5° (81 model grid points), and panels (c) and (f) to an average of 5°×5° (324 model grid points).

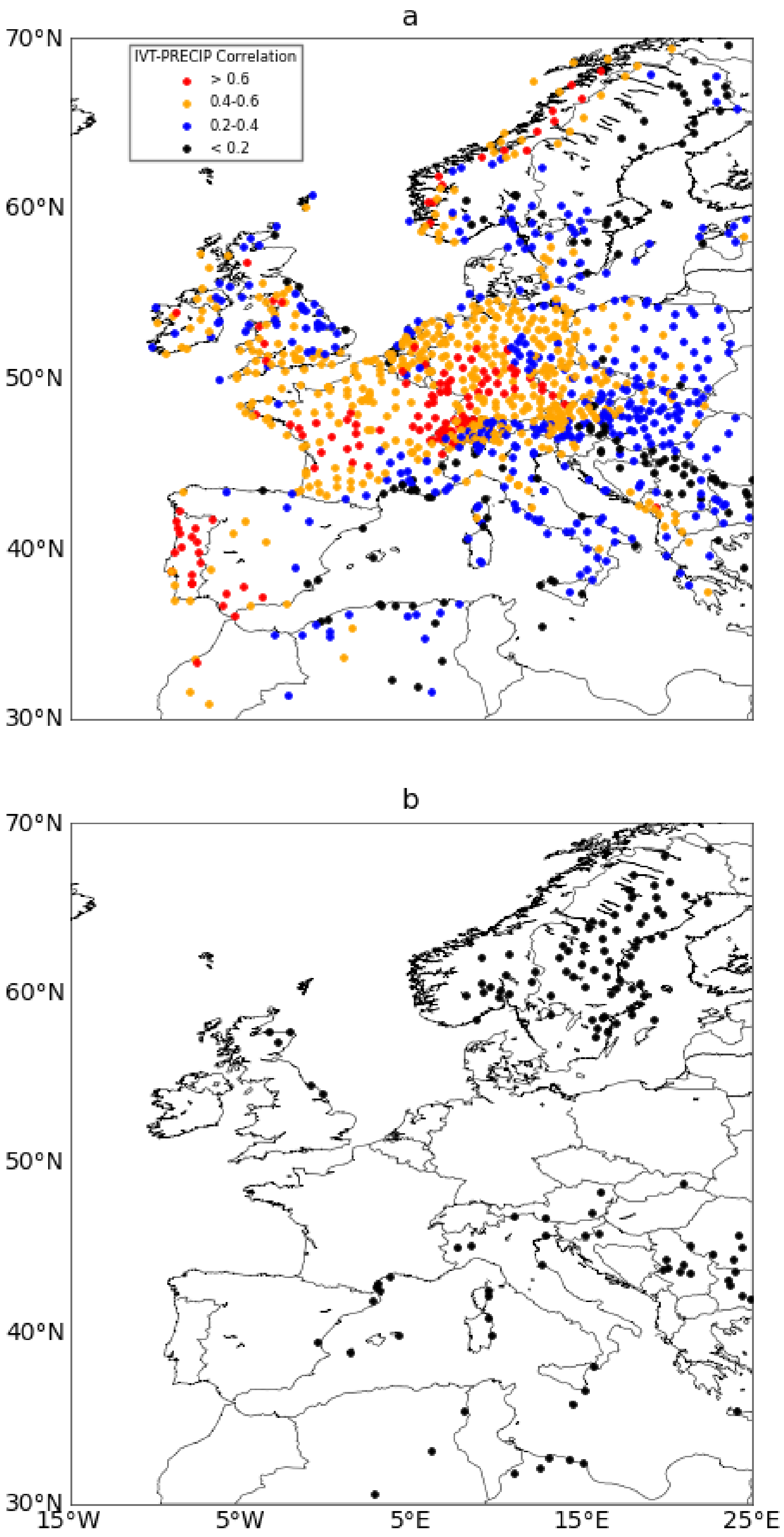

a
IVT-PRECIP Correlation
> 0.6
0.4-0.6
0.2-0.4
< 0.2
70°N
60°N
50°N
40°N
30°N
b
70°N
60°N
50°N
40°N
30°N
15°W
5°W
5°E
15°E
25°E

Supplementary Figure 2: Linear correlation between time series of (square root transformed) daily precipitation totals and daily-averaged water vapour transport from the closest ERA-Interim reanalysis grid point. Panel (a) shows stations with significant correlations at the 0.01 level; panel (b) shows stations with non-significant correlations (at the 0.01 level). The analysis is for the extended winters (December, January, February, and March) of 2009/10 to 2013/14 (five winters), which is equal to 606 days, and only stations with more than 50% of daily availability were considered.
